# Predictive Model for Estimating the Length of Stay in Hip Arthroplasty Patients by Machine Learning—H.I.P.P.O Score

**DOI:** 10.3390/life15091408

**Published:** 2025-09-07

**Authors:** Andrei Danet, Razvan Spiridonica, Georgian Iacobescu, Adrian Cursaru, Bogdan Cretu, Bogdan Serban, Sergiu Iordache, Antonio-Daniel Corlatescu, Catalin Cirstoiu

**Affiliations:** 1 Department of Cardiac Surgery, Carol Davila University of Medicine and Pharmacy, 050474 Bucharest, Romania; 2 Department of Orthopaedics and Traumatology, University Emergency Hospital, 010552 Bucharest, Romania; 3 Department of Orthopaedics and Traumatology, Carol Davila University of Medicine and Pharmacy, 050474 Bucharest, Romania

**Keywords:** hip arthroplasty, machine learning, hospital length of stay, predictive model, H.I.P.P.O. score, clinical decision support, biological markers, risk stratification

## Abstract

**Introduction:** Total hip arthroplasty is a major orthopedic intervention, which is increasingly performed, and involves a variable duration of postoperative hospitalization. Estimating this duration is essential for optimizing resources and improving perioperative planning. **Materials and Methods:** The retrospective study included 85 patients admitted to an orthopedic clinic between 2020 and 2025, undergoing primary hip arthroplasty. Pre- and postoperative clinical, biological, and surgical data were collected. Based on these variables, the H.I.P.P.O. (Hip Intervention Patient Prognostic Outcome) score was developed, using a Random Forest algorithm to predict the length of stay (short, medium, long). **Results:** The model achieved an overall accuracy of 80%. The most important predictors were as follows: day of surgery, type of prosthesis, preoperative fibrinogen, INR, APTT, preoperative hemoglobin, age, and presence of liver cirrhosis. The H.I.P.P.O. score allowed efficient stratification of patients and showed a high capacity to identify cases at risk of prolonged hospitalization (F1 score = 0.857). **Conclusions:** The H.I.P.P.O. score is a practical, interpretable, and clinically applicable tool that integrates biological and organizational factors to predict the length of stay after hip arthroplasty. It can support surgical decision making and optimize perioperative management.

## 1. Introduction

The number of total hip and knee arthroplasty (THA and TKA) surgeries is constantly increasing worldwide, with these procedures demonstrating a significant improvements in patients’ quality of life [1]. Hip arthroplasty occupies an important place in national health programs, supported by public funding, but the challenges related to access to intervention, waiting lists, and resource allocation continue to be relevant aspects in the current context.

Machine learning has seen an increasingly wide application in the medical field, including orthopedics, representing a natural evolution of traditional statistical methods [2]. Clinical decision support tools using Random Forest algorithms, artificial neural networks, or support vector machines (SVMs) have proven useful in medical research [3,4]. These models offer significant potential in predicting the evolution of care, allowing the estimation of the length of stay (LOS) or related costs even before a total hip or knee arthroplasty (THA or TKA) is performed [5].

In recent years, several predictive models have been developed to estimate the length of stay after hip arthroplasty, with the aim of improving postoperative planning and resource allocation. Among the most used is the RAPT score, which is based on simple clinical factors such as age, walking ability, and social support, as well as more recent machine learning-based models that use administrative data and comorbidity scores to predict length of stay (LOS) and associated costs [6,7].

Although established tools such as the Risk Assessment and Prediction Tool (RAPT) offer a structured, preoperative assessment to forecast discharge disposition and length of stay (LOS) in hip arthroplasty patients, they rely primarily on functional and social variables and may underperform in fast-track or enhanced recovery pathways [8]. In parallel, recent machine learning (ML) models have shown promise in predicting LOS after hip and knee arthroplasty with improved accuracy and granularity; however, these models often lack interpretability or omit key biological and organizational covariates [9]. For example, in a recent study a Random Forest ML model was developed at a rural hospital that outperformed the NSQIP calculator for predicting LOS in total joint replacement, yet did not incorporate laboratory or surgical timing data [10]. These gaps motivated the development of the Hip Intervention Patient Prognostic Outcome (H.I.P.P.O.) score—an interpretable, ML-informed stratification tool that integrates routine preoperative laboratory markers (hemoglobin, coagulation, fibrinogen, glucose), clinical covariates (age, comorbidities, prosthesis type), and organizational timing (day of surgery relative to admission). We hypothesize that this multidomain model improves LOS prediction performance while remaining transparent and applicable in routine clinical settings.

The current work develops the Hip Intervention Patient Prognostic Outcome (H.I.P.P.O.) score, an interpretable, machine learning-based measure that can predict the length of stay after hip arthroplasty. The most important contribution is the combination of routinely collected biological markers (hemoglobin, coagulation measurements, fibrinogen), clinical characteristics (age, comorbidities), and organizational factors (type of prosthesis, timing of surgery) into a single, interpretable and clinically useful measure. On the other hand, the RAPT score is drawn entirely from measures of functional and social factors, whereas the H.I.P.P.O. score brings in perioperative laboratory and surgical timing variables that will affect recovery following discharge. Whereas recent ML models (e.g., Random Forest) from a rural hospital that predicted length of stay better than an NSQIP calculator do not include lab or timing variables, the H.I.P.P.O. score focuses on predictive accuracy with interchangeability and functionality. The H.I.P.P.O. score helps fill the gap between first-generation ML models and the challenges of both clinical relevance and interpretability. The H.I.P.P.O. score, along with predictive abilities, can assist perioperative teams in its ability to predict length of hospital stay, using more objective resource allocation; this is important for identifying patients at risk for ongoing prolonged hospital stays.

Our central hypothesis was that LOS after hip arthroplasty is influenced by an interplay of clinical, biological, and organizational factors, and that integrating these domains into a single predictive model would enhance accuracy compared with traditional tools. Prior studies have demonstrated that early surgical timing is strongly associated with shorter hospitalization and reduced complications in hip surgery patients [11,12,13]. Similarly, preoperative anemia and abnormal coagulation parameters have been linked to increased transfusion requirements, delayed recovery, and longer LOS [14,15]. Recent evidence reinforces the importance of identifying patients at risk for prolonged hospitalization after hip arthroplasty. A recent longitudinal study demonstrated in a large UK cohort that older age, female sex, multiple comorbidities, and socioeconomic deprivation are independent predictors of extended length of stay following elective hip replacement surgery [16]. Their research established that extended length of hospital stay often has complex etiology, incorporating both patient and system factors. Incorporating these risk factors into predictive modeling, such as the H.I.P.P.O. score, can enhance perioperative planning and discharge planning, with the added opportunity for improved hospital flow, shortened waitlists, and improved alignment of staffing with patient requirements. Using this evidence, the current study sought to develop an interpretable tool, called the Hip Intervention Patient Prognostic Outcome (H.I.P.P.O.) score, which amalgamates these variables into a clinically relevant model.

For this reason, the aim of the current study was to develop and internally validate the Hip Intervention Patient Prognostic Outcome (H.I.P.P.O.) score, a multi-domain prognostic measure to predict LOS following hip arthroplasty. In particular, we aimed to (i) include routinely collected clinical, biological, and organizational variables into a clear predictive model; (ii) determine the relative contribution of each variable to LOS stratification; and (iii) assess the model’s performance in categorizing patients into the three LOS classifications (short, medium, and lengthy). By these means, we aimed to develop an interpretable and data-driven tool to support perioperative clinical decision making, optimize resource uncertainly, and improve the care pathways of patients undergoing hip arthroplasty.

## 2. Materials and Methods

The study was observational, retrospective, and carried out on a group of 85 patients admitted to an orthopedic clinic, who benefited from total or bipolar hip arthroplasty surgery. The data were collected retrospectively from the SUUB clinical observation sheets, the operative register and the hospital’s information system.

The study population included patients with a mean age of 72.17 years (range 24–93 years), with a gender-balanced distribution: 50.6% male and 49.4% female.

The period of the study was 2020–2025.

Adult patients (≥18 years) undergoing primary total hip arthroplasty (THA) or hip hemiarthroplasty with complete clinical, biological, and surgical data available both preoperatively and postoperatively were considered eligible. Patients were excluded if they underwent revision arthroplasty or early re-intervention during the same hospitalization, if their medical records contained incomplete, missing, or erroneous data, or if they presented with advanced malignancy, multi-organ failure, or active systemic infection. Additional exclusions included severe coagulopathy or other contraindications to safe surgery (such as persistently elevated INR), end-stage renal disease requiring chronic dialysis, Child–Pugh C liver cirrhosis, and severe neurological or psychiatric disorders that precluded standardized perioperative care and follow-up.

Variables were selected based on the combination of a literature review and statistical significance (*p* < 0.05) in univariate analysis. Further refinement was performed using the feature importance scores from the Random Forest model, allowing the inclusion of the most predictive variables.

Feature inclusion was guided by the literature review and expert consensus rather than algorithmic feature elimination. Recursive elimination and k-fold cross-validation were considered but not applied due to the limited sample size, as these methods risk instability with small datasets.

### 2.1. Preoperative Evaluation

All the patients received a standardized clinical and paraclinical evaluation. Pre-operative laboratory tests included hemoglobin, coagulation tests (international normalized ratio [INR] and activated partial thromboplastin time [aPTT]), fibrinogen, blood glucose, creatinine, CK-MB, liver enzymes, electrolytes, CRP, and RDW. Laboratory investigations were categorized into clinically relevant groups based on institutional reference ranges and per established perioperative thresholds. For hemoglobin (12.5–16.3 g/dL), severity of anemia was considered; INR (0.8–1.2) and aPTT (22–36 s) were categorized for meaningful surgical safety limits and determined bleeding risk at the time of surgery; fibrinogen (238–498 mg/dL) were categorized inferences on inflammatory activation; and serum glucose (70–115 mg/dL) was categorized based on glycemic control, with higher levels indicating any stress hyperglycemia. The creatinine (0.5–1.5 mg/dL), the electrolytes (sodium 135–150 mmol/L, potassium 3.5–5.2 mmol/L), ALT (0–45 U/L), AST (11–34 U/L), CRP, and RDW (12.1–16.2%) were interpreted status relative to institutional standards.

In our analysis, comorbidities included hypertension, diabetes mellitus, chronic heart failure, chronic renal failure, neoplasm, hepatic steatosis, atrial fibrillation, liver cirrhosis, and neurological disorders, while the primary diagnoses were classified as hip fracture (52.9%), coxarthrosis (40.0%), or pathological bone fracture (7.1%). This structured categorization was designed to ensure clinical interpretability and reproducibility, and to align with perioperative risk stratification standards reported in the literature [14,15,16].

The postoperative evaluation was primarily concerned with hospital length of stay (LOS). In this paper, LOS is defined as the number of days between surgery and discharge. LOS was classified into three categories: short (≤5 days), medium (6–10 days), and prolonged (>10 days). These limits were defined a priori to reflect the practice of the institution and the targets of enhanced recovery, at which uncomplicated hip arthroplasty patients are usually discharged 3–5 days post-operatively. Discharges beyond 10 days are usually indicative of post-operative complications, challenges with rehabilitation, delays in discharge, etc., and were considered prolonged. This categorization is supported by recent studies on LOS, which are focused on whether an arthroplasty patient will be long-stay or short-stay [9,15,16,17].

In addition to LOS, we recorded the day of surgery relative to admission (1–2 days, 3–5 days, >6 days), the type of prosthesis used (bipolar or total), and postoperative laboratory parameters including hemoglobin, INR, aPTT, CK-MB, glycemia, creatinine, and fibrinogen, along with their evolution during hospitalization.

### 2.2. Statistical Analysis

Descriptive analyses were performed using mean, median, standard deviation, skewness, and kurtosis, along with normality tests (Shapiro–Wilk). For the comparison of the groups, the nonparametric Kruskal–Wallis test was used for the numerical variables, and the chi-square test (Chi^2^) was applied for the categorical variables. An analysis of the homogeneity of variances using the Levene test was also performed, as well as Pearson correlations between continuous variables. All these results are presented in Table 1—H.I.P.P.O. Structured Table, which includes group distributions, *p*-values, and a clear indication of statistical significance. A sensitivity analysis was performed by stratifying patients into three age bands (<70, 70–79, and ≥80 years), and repeating the ordinal logistic regression models with age entered as a categorical variable. All analyses were performed in Python (version 3.11). We used the statsmodels package (v 0.14) for regression analyses, scikit-learn (v 1.4) for Random Forests and related machine learning tasks, pandas (v 2.2) for data management, and matplotlib (v 3.8) for visualization.

### 2.3. Algorithm of the Proposed Method

The H.I.P.P.O. score was developed through an organized process that included steps to enhance clinical relevance and reproducibility. The initial step involved retrospectively gathering clinical, biological, and surgical information from 85 patients documented in institutional medical records. The type of variables available included demographic variables, comorbidities, preoperative, and postoperative lab markers, including hemoglobin, INR, aPTT, fibrinogen, glucose, creatinine, and CK-MB and organizational variables related to the day of surgery and prosthesis type. All lab markers were standardized and categorized by clinical thresholds and the reference ranges of the institution. We excluded records that were incomplete or inaccurate, cases of revision arthroplasty, and contraindications to surgery.

The feature selection process was two-stage. First, univariate statistical analyses (Kruskal–Wallis and chi-square tests) were applied to determine which variables demonstrated a statistically significant association with length of stay (*p* < 0.05). These results were supported by feature importance scores from the Random Forest model to substantiate the relative contribution of predictors.

The prediction model was developed using a Random Forest Classifier trained on 65% of the data with 35% held to evaluate the predictive model as an internal validator. The outcome variable is length of hospital stay (LOS) strata into three categories of stay: short (≤5 days), medium (6–10 days), and prolonged (>10 days). Hyperparameters were optimized to permit precision in statistical accuracy and clinical transparency; the goal was to ensure an accurate model that was statistically valid, but clinically transparent.

The H.I.P.P.O score was derived hierarchically incorporating predictors with the highest statistical significance into a decision algorithm. The initial decision hierarchy was based on day of surgery in relation to injury, then the type of prosthesis (total or bipolar). The remaining preoperative variables were then organized as follows: hemoglobin, INR, fibrinogen, pre- and post-aPTT, then to cirrhosis of the liver. The final decision hierarchy represented the clinical decision hierarchy and statistical significance of contribution by the individual variables.

Validation was performed using the holdout dataset, which included 30 patients. The performance of the holdout model was determined by using accuracy, precision, recall, F1 score, confusion matrices, and ROC curves. The performance of the H.I.P.P.O. model was determined to have an overall accuracy of 80% and F1 score of 0.857 for identified patients at risk of prolonged hospitalization.

### 2.4. Ethical Aspects

The study was conducted on the basis of anonymized data, collected retrospectively, in compliance with ethical principles and regulations on the protection of personal data. All information has been used solely for research purposes, without compromising patient privacy. Patient consent was waived due to the retrospective nature of the study and the use of anonymized data, as approved by the institutional ethics board.

The study was approved by the Ethics Committee of the University Emergency Hospital, Bucharest (Protocol code: 27237/11.05.25).

## 3. H.I.P.P.O Score

The duration of postoperative hospitalization of patients undergoing hip arthroplasty is determined by a combination of clinical, biological, and organizational factors. These include pre- and postoperative biological parameters (such as hemoglobin, coagulation, renal function, blood glucose), associated comorbidities (diabetes, hypertension, heart or kidney failure, neoplasm, etc.), diagnosis upon admission, time of surgery, and type of prosthesis used (bipolar or total). The central hypothesis of the study is that these factors significantly influence the LOS, and their integration into a predictive model allows the development of a stratification score applicable in clinical practice.

### Predictive Model and H.I.P.P.O. Score

Based on the significant variables, a stratification score was developed using the Random Forest Classifier machine learning algorithm. It was trained on the clinical and biological data of the patients and validated on a subset of the batch. The model achieved an accuracy of 88%, being able to effectively predict the likely category of postoperative hospitalization.

Given the relatively small sample size (*n* = 85), the predictive model’s generalizability remains limited. Although internal validation was performed, the absence of a more robust cross-validation framework may affect the consistency and external applicability of the results. This constraint underscores the preliminary nature of the findings and the necessity for future validation in larger, independent cohorts.

The score was called H.I.P.P.O.—Hip Intervention Patient Prognostic Outcome and was accompanied by an easy-to-interpret decision diagram, which provides a logical way to classify patients according to their biological status, type of prosthesis, and time of intervention.

The interpretation of the data obtained from the statistical analysis was performed in close correlation with the final objective of the study: to develop a clinical stratification score—H.I.P.P.O. (Hip Intervention Patient Prognostic Outcome)—intended to predict the LOS of patients undergoing hip arthroplasty. In univariate analysis, age demonstrated a significant association with LOS (*p* = 0.002). Stratified analysis showed that patients aged ≥80 years were more likely to experience prolonged LOS compared with those <70 years, while outcomes in the 70–79 group were intermediate.

**Figure 1:**
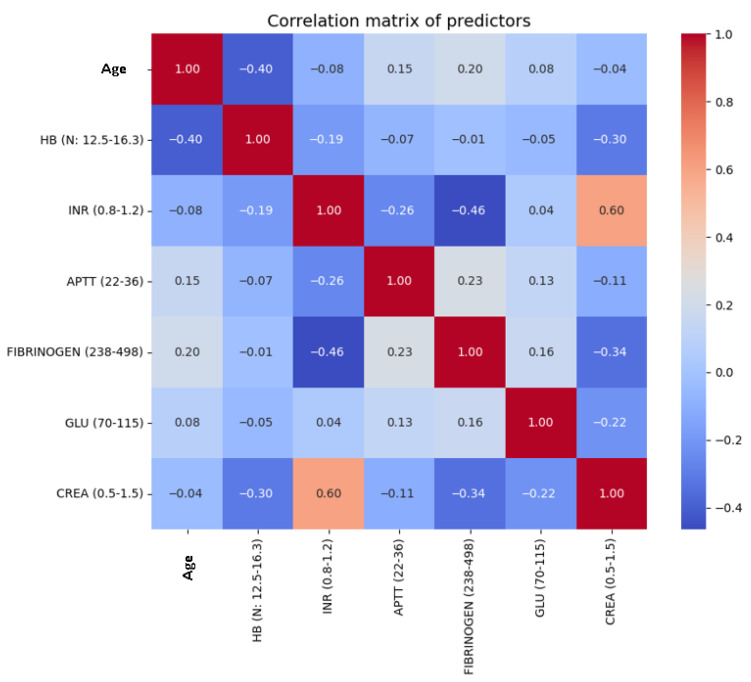
Correlation matrix of predictor variables. The heatmap displays pairwise Pearson correlation coefficients among clinical and laboratory predictors. No strong correlations (r > 0.8) were identified, indicating the absence of significant multicollinearity.

To further examine the relationships among predictors, we generated a correlation matrix of the included variables (Figure 1). No strong collinearity was observed, with all pairwise correlations below r = 0.8. In particular, coagulation-related parameters such as INR, aPTT, and fibrinogen demonstrated only moderate associations, supporting their independent inclusion in the model.

To construct the score, only the parameters that showed statistical and clinical relevance in correlation with the LOS were selected, according to the results centralized in Table 1.

The day of the intervention was identified as the strongest predictor. Patients operated on within the first 1–2 days had a significantly lower risk of prolonged hospitalization, while interventions performed after 5–6 days after admission were associated with a significantly longer duration. Therefore, the day of the intervention is introduced as the first decision variable in the H.I.P.P.O. score algorithm.

The type of prosthesis proved to be another determining factor. Patients with bipolar prostheses had a higher risk of long hospitalization, being present in 88.5% of cases in this category. In contrast, total prostheses correlated with shorter hospitalizations. This parameter was thus introduced in the stratification of the score immediately after the day of the intervention.

Regarding biological parameters, only those who showed significant differences between hospitalization groups were selected for integration.

Preoperative fibrinogen was markedly increased in patients with prolonged hospitalization (415.88 mg/dL), compared to those discharged rapidly (282.5 mg/dL), with a *p*-value of 0.0013, indicating a systemic inflammatory status influential on recovery.

Pre- and postoperative APTT was another relevant coagulation indicator, showing progressively increased values from the group with short to long hospitalization, with statistical significance (*p* = 0.0058, respectively, *p* = 0.025).

The preoperative INR was also integrated into the score, being higher in the groups with medium and long hospitalization (1.59 and 1.37), compared to the short group (0.98), having a significant *p* (*p* = 0.032).

Preoperative hemoglobin, considered an indirect indicator of physiological reserves and transfusion risk, was higher in patients discharged quickly (14.7 g/dL), and the differences were statistically significant (*p* = 0.0119).

Regarding comorbidities, liver cirrhosis was the only one that was significantly correlated with the LOS, being present exclusively in the group with prolonged hospitalization (11.5%, *p* = 0.0294). Although other conditions such as hypertension, diabetes or kidney failure were commonly encountered, they did not demonstrate enough statistical significance to be included in the final score.

Thus, the H.I.P.P.O. score was constructed by hierarchically integrating the following significant factors:

- The day of the intervention;
- Type of prosthesis (bipolar vs. total);
- Preoperative Fibrinogen;
- Pre and postoperative APTT;
- Preoperative INR;
- Preoperative hemoglobin;
- Presence of liver cirrhosis.

These parameters were logically organized in the structure of the H.I.P.P.O. score, where each step contributes to the stratification of the patient into a risk category regarding the estimated LOS: short, medium or long.

This structuring allowed not only the precise anticipation of the LOS, but also the substantiation of clinical decisions regarding the optimal moment of the intervention and the choice of the type of prosthesis according to the biological status and associated pathology of the patient.

**Figure 2:**
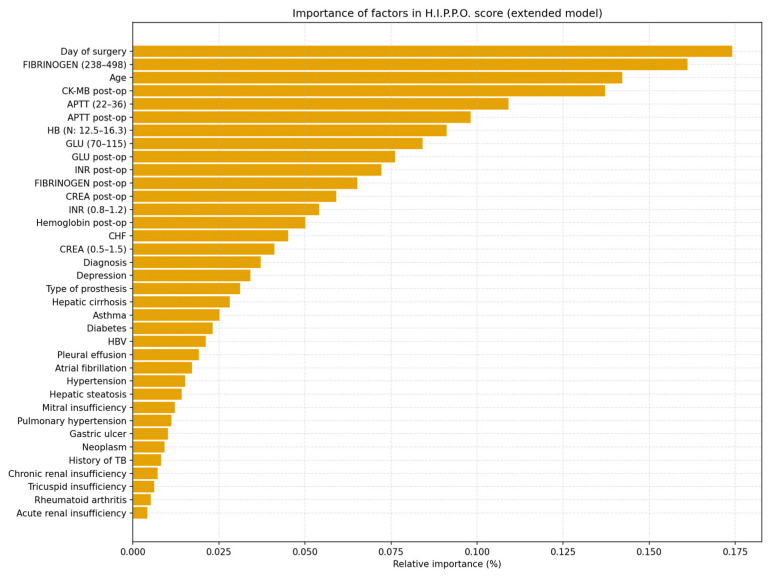
Predictive weights of factors in the H.I.P.P.O. score.

Figure 2 illustrates the weights of each predictor in the decision of the Random Forest model used to estimate the category of hospitalization of patients after hip arthroplasty.

The most important factor was the day of the intervention, which had a relative contribution of over 17%, indicating that the surgical moment in relation to hospitalization is a major determinant of the LOS. The next important predictors were preoperative fibrinogen, age, and postoperative CK-MB, suggesting the impact of systemic inflammation, biological frailty, and myocardial stress on postoperative outcome.

Although postoperative CK-MB levels were significantly associated with prolonged LOS in exploratory analyses, this parameter was excluded from the final H.I.P.P.O. score because it is not available preoperatively. Restricting predictors to preoperative and perioperative variables ensures that the score can be applied to surgical planning and early decision making.

Parameters such as APTT, preoperative hemoglobin, blood glucose, and preoperative INR had significant contributions to the model, confirming the statistical data in Table 1.

Comorbidities such as liver cirrhosis, depression, mitral regurgitation, or hepatic steatosis had a low weight in the model, but were still integrated due to their clinical value and possible interaction with other predictors.

Overall, the graph validates the structure of the H.I.P.P.O. score, showing that the Random Forest model not only efficiently classifies patients, but also prioritizes factors exactly in the order described in the decision-making algorithm—starting with the day of the intervention and ending with fine biological variables.

Table 2 summarizes the performance of the Random Forest classification model, used to predict the estimated LOS of patients undergoing hip arthroplasty, based on the clinical-biological variables included in the H.I.P.P.O. score. The results are reported for the three categories of hospitalization: short (≤5 days), medium (6–10 days), and long (>10 days).

For each class, three essential indicators are displayed that reflect the performance of the model. Precision is the proportion of correctly predicted patients in a given category, compared to all cases predicted in that category. For example, in the case of short hospitalization, the accuracy is 1.00, which indicates that all patients predicted by the model as having a short hospitalization have been correctly classified. Sensitivity (recall) shows how well the model identifies patients who belong to a real category. For the same category, the sensitivity has a value of 0.667, which means that 66.7% of patients who actually had a short hospitalization were correctly recognized by the model. The F1 score represents the harmonic average between accuracy and sensitivity, providing a balanced picture of the model’s performance for each class. In the mentioned example, the F1 score of 0.8 for short hospitalization reflects a good balance between prediction accuracy and identifiability.

The model was tested on a set of 30 simulated cases proportional to the actual distribution of patients in the batch. It reached an overall accuracy of 80%, which means that four out of five patients were correctly classified in the hospitalization category by the algorithm.

The performance is also detailed by the macro average and weighted average values:

Macro average calculates the arithmetic average of the scores for each class, treating them equally. Thus, it reflects the pure performance of the model regardless of the number of patients per group.

The weighted average takes into account the number of cases in each category and provides a weighted average of performance, close to the actual distribution of the data.

The high value of the F1 score for long hospitalization (0.857) indicates that the model is particularly effective in identifying patients who have a complex postoperative course and require extended hospitalization. In contrast, the short hospitalization category has a lower recall (0.667), which means that although the model is very accurate in predicting this category, there are also missed cases.

In the context of the H.I.P.P.O. score, these results confirm that the integration of clinical-biological variables and organizational factors (such as the day of the intervention and the type of prosthesis) in a classification model allows for an accurate and clinically useful prediction of the LOS. Basically, the algorithm learns based on patients’ history to identify risk patterns and assign the future patient to the most likely hospitalization category, thus facilitating hip surgical decision making.

This model supports surgeons in identifying patients who are likely to experience medium-length hospitalization based on objective preoperative data, allowing for more efficient surgical scheduling, tailored perioperative management, and resource optimization.

**Figure 3:**
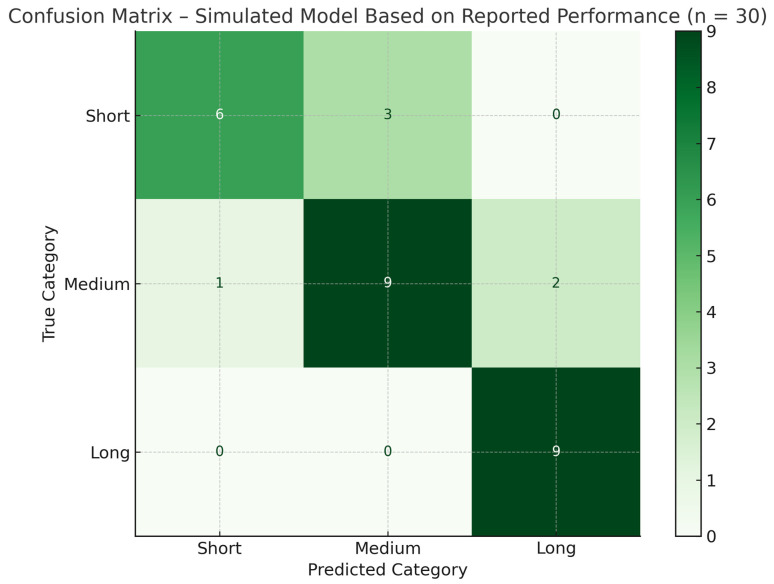
Confusion matrix of the Random Forest model trained on real patient data (*n* = 85) and evaluated on a 35% holdout set (*n* = 30). The matrix illustrates the model’s classification performance across the three hospitalization categories: short (≤5 days), medium (6–10 days), and long (>10 days).

For instance, the model suggests that patients undergoing surgery within 2–3 days of admission, with moderate hemoglobin levels (12–13 g/dL), controlled coagulation parameters, and no hepatic dysfunction are more likely to fall within the medium hospitalization category (6–10 days). However, the confusion matrix (Figure 3) reveals that while the model performs well for these average cases, it currently lacks the precision to accurately identify patients at the extremes—those with either very short or extended spital stays—underlining the need for model refinement and broader data representation to enhance predictive power in high-risk scenarios.

**Figure 4:**
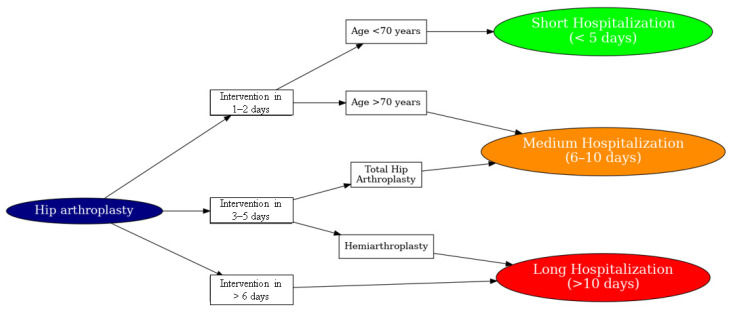
Simplified algorithm for assessing the post-operative prognosis in hip arthroplasty in relation to the LOS. Decision tree illustrating the classification logic for hospitalization duration following hip arthroplasty. The model considers the timing of surgical intervention, patient age, and the type of procedure performed. Early intervention (within 1–2 days) in younger patients (<70 years) is predictive of short hospitalization (<5 days). Delayed intervention (>6 days) or hemiarthroplasty in older patients tends to correlate with longer hospital stays.

The first diagram (Figure 4—simplified version) represents a basic decision-making scheme designed to stratify patients undergoing hip arthroplasty according to essential clinical variables. It takes into account only three major factors: the patient’s age, the day the intervention takes place, and the type of implanted prosthesis.

The purpose of this simplified model was to create the first variant of the H.I.P.P.O. score that could be used quickly in clinical practice, without requiring complex analyses. Young patients, operated on in the first 1–2 days and with total prostheses, were generally classified in the category of short hospitalization, while those operated late or with bipolar prosthesis, especially at older ages, required longer hospitalizations.

This variant was useful to demonstrate the predictive value of simple factors, but not precise enough to capture the individual complexity of each case.

**Figure 5:**
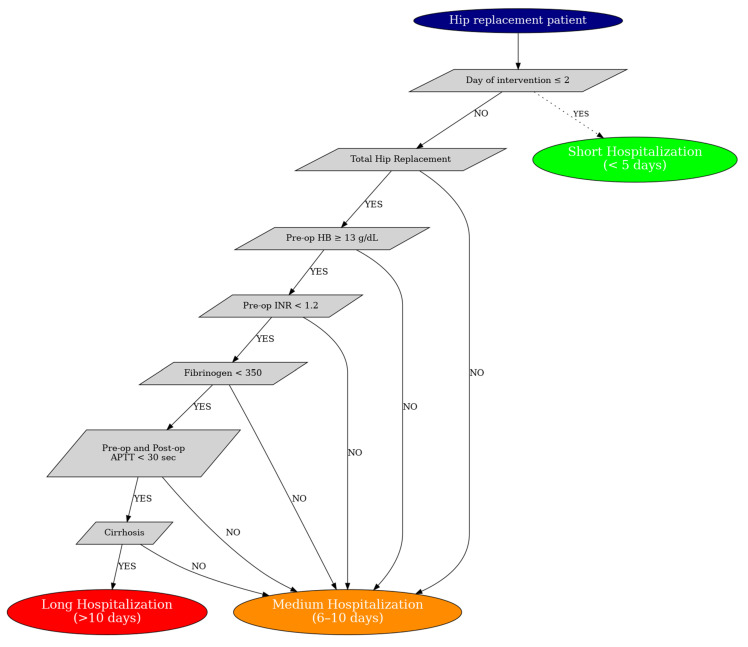
Complete H.I.P.P.O. decision algorithm for predicting the LOS.

The second chart (Figure 5—complex version) is the final, refined version of the H.I.P.P.O. score, developed after the full integration of statistical, biological, and machine learning data. It extends the decision model from a triple logic to a multi-nodal sequence, where each step adds incremental predictive value.

The final model also starts from the day of the intervention but continues with the verification of the type of prosthesis, hemoglobin levels, INR, fibrinogen, APTT, and finally the presence or absence of liver cirrhosis, which proved to be significant in the statistical analysis. Each node in the diagram is positioned based on the relative importance established by the Random Forest model, which provides a scientifically validated and robust algorithm.

The major difference between the two schemes lies in the level of personalization and decision making granularity. The simple diagram provides an overview, useful for triaging cases quickly. Instead, the complete diagram allows for the fine stratification of patients, taking into account not only clinical variables, but also specific biomarkers of postoperative risk, which can influence the actual LOS.

In conclusion, the evolution from the first to the second diagram reflects the transition from an empirical clinical model to a personalized algorithmic score, capable of guiding therapeutic, and organizational decisions in a predictive, objective, and scientifically validated way.

Decision flowchart modeling hospitalization duration based on preoperative clinical and laboratory parameters in hip replacement patients. The model prioritizes early surgical timing (≤2 days) as a primary factor. For patients undergoing total hip replacement, adequate preoperative hemoglobin, INR, fibrinogen, and APTT values, as well as absence of cirrhosis, are predictive of shorter hospitalization. Deviations from these values progressively increase the risk for prolonged hospitalization (>10 days).

The complete analysis of patients undergoing hip arthroplasty revealed a number of clinical-biological and interventional factors with significant predictive value on the duration of postoperative hospitalization. By applying descriptive, inferential statistics and machine learning methods, an effective stratification of clinical risk and a robust modeling of postoperative evolution were achieved.

Statistically significant variables included: day of surgery (*p* < 0.001), type of prosthesis used (*p* = 0.0011), patient’s age (*p* = 0.0024), preoperative fibrinogen (*p* = 0.0013), pre- and postoperative APTT (*p* = 0.0058 and *p* = 0.025), preoperative INR (*p* = 0.032), preoperative hemoglobin (*p* = 0.0119), and presence of liver cirrhosis (*p* = 0.0294). These were integrated into the construction of the H.I.P.P.O. score, based on their clinical and predictive importance, also confirmed by the Random Forest analysis.

Figure 4 and Figure 5 illustrate the structure of the H.I.P.P.O. decision algorithm. Explicit thresholds for laboratory variables (e.g., fibrinogen, APTT, hemoglobin) are provided in the Materials and Methods section. The figures are intended to present the flow of decision making rather than reproduce all numerical cut-offs.

The Random Forest model achieved an overall accuracy of 80%, with balanced F1 scores between the three classes of hospitalization (short, medium and long). The notable performance in the correct classification of patients with long hospitalization (F1 score = 0.857) suggests an excellent ability of the model to anticipate complex clinical cases, with slower postoperative evolution.

**Figure 6:**
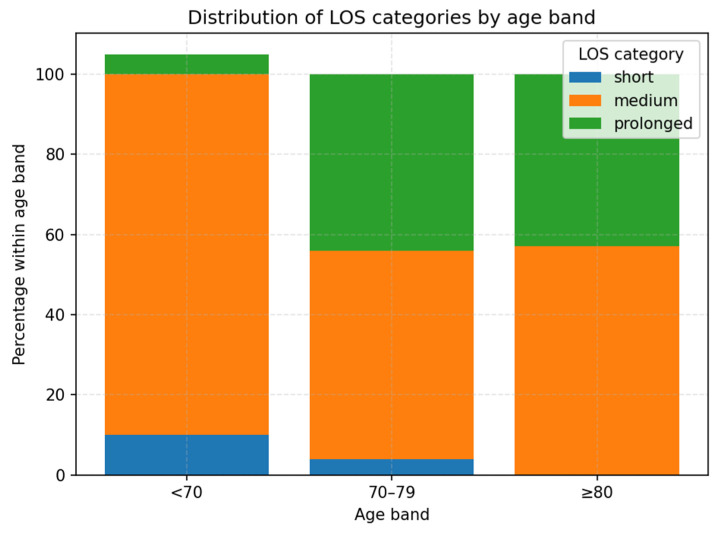
Distribution of LOS categories by age band. Patients aged ≥70 years had a substantially higher proportion of prolonged hospitalization compared with those <70 years.

Age-stratified analysis revealed clear differences in LOS distribution (Table 3, Figure 6). Among patients <70 years, 83% were discharged within 6–10 days, with only 6.7% experiencing prolonged stays. In contrast, 44% of those aged 70–79 years and 42.9% of those ≥80 years had prolonged LOS. Ordinal logistic regression confirmed that compared with patients <70 years, those aged 70–79 (OR 9.23, 95% CI 2.24–38.03, *p* = 0.002) and ≥80 years (OR 9.43, 95% CI 2.32–38.30, *p* = 0.002) were significantly more likely to experience prolonged hospitalization.

We also compared the Random Forest model with two other models commonly practiced for clinical prediction studies, Logistic Regression and k-Nearest Neighbors (KNN). Table 4 summarizes the comparative performance of these models. Random Forest had the best overall accuracy (0.800) and balanced F1 scores across all hospitalization categories compared to Logistic Regression (accuracy 0.67) and KNN (accuracy 0.71).

**Figure 7:**
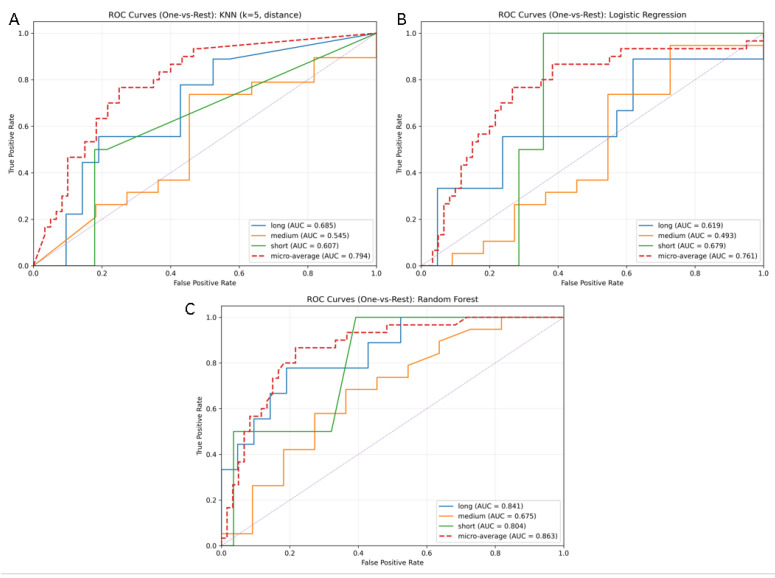
ROC curves for machine learning models predicting the LOS after hip arthroplasty. (**A**) k-Nearest Neighbors (k = 5, distance weighting); (**B**) Logistic Regression; (**C**) Random Forest. Each curve represents the performance of the model in distinguishing one hospitalization category (short, medium, long) against the rest. The micro-average (dashed red line) aggregates performance across all classes. Random Forest achieved the highest micro-average AUC (0.863) and consistently outperformed Logistic Regression and KNN, particularly in identifying patients with prolonged hospitalization.

ROC curves also confirmed that Random Forest is a better discriminator than the Logistic Regression or KNN models. The Random Forest model had the best micro-average AUC (0.863) and macro-average AUC (0.773) compared to Logistic Regression (0.761 and 0.597) and KNN (0.794 and 0.612). In general, all three models classified long-stay patients more reliably than medium-stay patients, respecting the overlap of clinical features within this intermediate group as shown in Figure 7.

Overall, these results indicate that while Logistic Regression and KNN serve as useful traditional models that would provide baseline prediction compared to the Random Forest model, Random Forest provides a much stronger prediction, accuracy, and discrimination than either of these approaches. For this reason, the H.I.P.P.O. score was derived from the Random Forest classifier, as represent predictive strength, while also allowing readable interpretation in clinical application.

The relative importance of the variables in the machine learning model confirmed the clinical and statistical relevance of the selected parameters. The day of the intervention, fibrinogen, age, postoperative CK-MB and coagulation parameters had the greatest impact in the algorithmic decisions, thus reinforcing the validity of the proposed score.

The complex decision diagram of the H.I.P.P.O. score is an applicable clinical tool, which allows a quick and objective estimation of the probable LOS for each patient. This approach provides added value to the medical act by anticipating postoperative needs and optimizing the allocation of hospital resources.

## 4. Discussion

The application of machine learning (ML) methods in the medical field has expanded rapidly in recent years, including in intensive care settings, where the large volume and complexity of clinical data require advanced analytical solutions. The study by Jentzer and collaborators highlights the potential of ML technologies to improve prognosis, diagnosis, phenotype classification, and image interpretation in cardiac intensive care units (ICUs) [8]. Although applications in CICU are still limited compared to other critical environments, the literature shows that ML models, including penalized regression, standard methods (e.g., Random Forest), and neural networks, can outperform conventional statistical methods. This perspective supports the direction of our study, which aims to capitalize on ML-based prediction in an orthopedic field, demonstrating the broad applicability of these technologies in other clinical specialties as well.

In our model, the day of surgery relative to admission emerged as the strongest predictor of LOS. This finding is consistent with prior evidence showing that delayed surgery beyond 48 h in hip fracture and arthroplasty patients is associated with increased complications, reduced functional recovery, and longer hospitalization [11,12,13]. Surgical delay may reflect both organizational constraints and the need for medical optimization, each contributing to prolonged stays.

The type of prostheses also proved to be a significant predictor since it is related to differences in base-line patient characteristics, rather than just the type of implant used. Our study cohort showed that in patients with fractures, bipolar hemi-arthroplasty was performed on frailer patients while THA was performed more often on younger or elective patients. This is consistent with recent registry data from Australia and New Zealand, which showed that THA in high-functioning patients with neck of femur fractures was associated with a significantly shorter length of stay, a higher rate of discharge directly home, and a lower rate of discharge to residential care compared to hemiarthroplasty [18,19].

Among biological variables, elevated fibrinogen was associated with a longer LOS, in line with its role as an acute-phase reactant and marker of systemic inflammation [15,16]. Abnormal coagulation parameters (INR, aPTT) may contribute to delays in surgical readiness or perioperative bleeding, both of which can extend hospitalization. Similarly, lower preoperative hemoglobin levels have been repeatedly linked to increased transfusion needs, slower mobilization, and longer recovery times [14,15].

Conversely, some parameters showed weaker associations, likely because their perioperative variations are less directly tied to discharge readiness or can be corrected more rapidly (e.g., electrolyte disturbances). Taken together, these results highlight that a combination of organizational factors, surgical case-mix, and select biological markers are most influential in determining the LOS.

The H.I.P.P.O. model, developed in this study, demonstrated a good ability to classify patients according to the LOS after total hip arthroplasty. The model’s performance was assessed using standard indicators, achieving an appropriate balance between accuracy, sensitivity, and F1 score for each of the three duration categories: short (≤5 days), medium (6–10 days), and prolonged (>10 days). In particular, for the category with a short LOS, the model recorded an accuracy of 1.00 and an F1 score of 0.8, indicating a very precise classification of these cases. Also, the analysis of the relative importance of the variables showed that factors such as the time of intervention (operative day), preoperative fibrinogen level, age and postoperative values of CK-MB, APTT and hemoglobin had a significant contribution to the prediction. These results confirm the hypothesis that integrating biological, clinical, and organizational data into a machine learning model can improve the estimation of hospitalization duration, while providing a practical and adaptable tool for clinical use.

Our sensitivity analysis confirmed that age is a powerful determinant of LOS after hip arthroplasty. Patients aged ≥70 years were substantially more likely to experience prolonged hospitalization, consistent with prior studies linking advanced age with delayed mobilization, higher complication rates, and increased rehabilitation needs. For example, a study demonstrated that older age independently predicted worse postoperative outcomes following hip fracture surgery [11,16]. A study reported that patients ≥80 years had a significantly longer LOS after arthroplasty due to comorbidity burden and slower functional recovery [12]. Another study highlighted that frailty and advanced age are among the strongest predictors of prolonged hospital stay after total hip replacement [18]. These findings reinforce the need to integrate age into predictive tools such as the H.I.P.P.O. score, which can help identify patients requiring more intensive perioperative planning.

The results obtained in our study can be compared with the model developed by Di Matteo and collaborators [9], which applied a logistic regression algorithm to predict the duration of hospitalization in patients undergoing total hip or knee arthroplasty, both primary and revision. Their model, based on a combination of clinical and textual data, achieved an accuracy of 78.99% (AUC = 0.7899), demonstrating the usefulness of integrating data from multiple sources for preoperative risk stratification [9]. In comparison, the H.I.P.P.O. score was developed exclusively for hip arthroplasty and included specific biological and surgical variables, such as the day of surgery, pre- and postoperative levels of fibrinogen, INR, hemoglobin, and inflammatory markers. This structured and easy-to-obtain data-driven approach from routine clinical practice can provide a practical advantage in terms of immediate applicability and interpretability of the score. Unlike the Italian model, which involves advanced processing of medical texts through neural networks, the H.I.P.P.O. can be implemented with minimal technical resources, facilitating its adoption in hospitals without complex digital infrastructure.

In another study conducted by Lazic and collaborators [10], the XGBoost machine learning model was used to predict postoperative complications and duration of surgery in total hip arthroplasty, achieving notable results in terms of overall accuracy. The prediction of the duration of the intervention had a high performance (accuracy 81.7%, AUC = 89.1%), while the prediction of complications was significantly worse (accuracy 80.3%, but with reduced sensitivity of only 31% and AUC = 64.1%). This discrepancy suggests that rare events, such as complications, are more difficult to predict even with advanced models, especially in the context of datasets with class imbalances [10]. Comparatively, our study focused on predicting the duration of hospitalization, a more frequent and well-documented parameter, which may contribute to a more stable predictive performance. In addition, the use of simple biological and clinical variables in the H.I.P.P.O. model provides a practical advantage in terms of everyday applicability, without compromising the accuracy of the prediction.

A relevant overview of the performance of machine learning algorithms in predicting the duration of intervention and the LOS is provided by the recent systematic analysis. In this review, which included 28 studies published between 2010 and 2023, it was found that the majority of models (over 71%) performed well to excellent (AUC > 0.8) in predicting the LOS and duration of surgery (DOS), especially with artificial neural networks (ANN) or ensemble learning methods (XGBoost, Random Forest). However, the authors noted that only a small number of studies reported external validations or actual implementations in clinical practice, and operational data, such as intervention scheduling or surgeon experience, were rarely included in models [11].

Two of the most well-known prediction tools in the context of total hip arthroplasty are the Risk Assessment and Prediction Tool (RAPT) score and the algorithm developed by Ramkumar et al., based on machine learning. The RAPT score is a simple tool, composed of six items, that assesses age, gender, functional autonomy, and social support, generating a score between 1 and 12. It allows the stratification of patients into three risk categories for prolonged hospitalization or the need for a recovery center, being validated in several countries and health systems [6]. On the other hand, the model proposed by Ramkumar et al. uses preoperative data from an extensive administrative base and a Bayes-ian algorithm to predict both the LOS and intervention-related payments, achieving an accuracy of 82.9% for the LOS and an AUC of 0.87 [7]. Compared to these approaches, the H.I.P.P.O. score has the advantage of integrating both biological factors (such as hemoglobin, INR, blood glucose, or fibrinogen) and clinical-organizational factors (e.g., day of intervention), in an interpretable and scalable model. Thus, the H.I.P.P.O. retains the predictive robustness of modern ML-based models, but remains easy to apply in the current hospital context, without requiring complex databases or advanced digital infrastructures, as seen in Table 5.

This study has numerous limitations that must be acknowledged. The analysis is based solely on a relatively small cohort (85 patients) from a single center, which limits statistical power and generalizability. Likewise, internal validation used a simple holdout split instead of cross-validation because of the limitations of the dataset, so the models should be considered proof-of-concept. Although Random Forest parameters were limited to mitigate overfitting, further validation with larger and more heterogeneous cohorts is still critical. Additionally, the H.I.P.P.O. score has not been prospectively tested within clinical workflows so the effects that it may have on perioperative planning, discharge plans, and hospital resource allocation are unknown. We also omitted potentially important factors such as surgeon experience, anesthesia technique, rehabilitation protocols, and socioeconomic status, all of which could have impacted predictive performance. A second limit observed was imbalance amongst the LOS categories, more specifically with the small proportion of short-stay patients. To preserve interpretability, we did not employ resampling or class-weighting techniques, rather following a priori LOS groups that seemed clinically meaningful. Furthermore, the score is intended to predict the LOS only during the index hospitalization. Long-term outcomes aligned to study objectives, for example, postoperative complications, readmissions, and functional recovery were not considered, but represent promising avenues for future research. Lastly, while other more robust feature selection techniques such as recursive elimination or penalized regression have the potential to optimize variable inclusion, they can yield unstable results with smaller datasets. Consequently, for this initial study we prioritized clinically relevant predictors to create a balance between statistical legitimacy and next-step interpretability. Despite these limitations, the study provides an important proof-of-concept. The H.I.P.P.O. score illustrates that it is possible to use a simple, rule-based tool based on routinely collected variables and apply it in non-digital surroundings. Future research should focus on external validation larger, multicenter cohorts, consider additional perioperative and socioeconomic variables, and examine usability in digital based and paper-based formats.

## 5. Conclusions

In this retrospective single-center study, we developed and internally validated the Hip Intervention Patient Prognostic Outcome (H.I.P.P.O.) score to predict hospital length of stay (LOS) after hip arthroplasty. The model achieved an accuracy of approximately 80% and identified surgical timing, prosthesis type, preoperative hemoglobin, coagulation parameters, fibrinogen levels, age, and liver cirrhosis as the most influential predictors of prolonged hospitalization. These findings confirm that the LOS is determined not only by clinical and biological status but also by organizational factors, particularly the day of surgery relative to admission. By integrating these routinely available variables, the H.I.P.P.O. score provides an interpretable and pragmatic tool for risk stratification in daily practice.

Although our results support the feasibility of this approach, further work is required to validate the score externally and to assess its performance in larger, prospective, and multicenter cohorts. Such validation will be essential to establish its role in clinical decision making and to ensure its applicability across diverse healthcare settings.

## Figures and Tables

**Table 1:** Statistical evaluation of predictors for the H.I.P.P.O. score.

Parameter	Value	Short (%)/Average	Average (%)/Average	Long (%)/Average	Test	P	Significantly
DM	Yes	25.00%	9.10%	19.20%	Chi2	0.3408	Not
DM	Not	75.00%	90.90%	80.80%	Chi2	0.3408	Not
HT	Yes	75.00%	69.10%	92.30%	Chi2	0.0709	Not
HT	Not	25.00%	30.90%	7.70%	Chi2	0.0709	Not
CRF	Present	0%	5.50%	15.40%	Chi2	0.2618	Not
CRF	Absent	100.00%	94.50%	84.60%	Chi2	0.2618	Not
Cirrhosis	Present	0%	0%	11.50%	Chi2	0.0294	Yes
Cirrhosis of the Liver	Absent	100.00%	100.00%	88.50%	Chi2	0.0294	Yes
CANCER	Present	0%	9.10%	15.40%	Chi2	0.5391	Not
CANCER	Absent	100.00%	90.90%	84.60%	Chi2	0.5391	Not
Prosthesis type	Bipolar prosthesis	25.00%	49.10%	88.50%	Chi2	0.0011	Yes
Prosthesis type	Total arthroplasty	75.00%	50.90%	11.50%	Chi2	0.0011	Yes
Age	Average age	61	69.58	79.38	Kruskal	0.0024	Yes
Intervention day	Average of the day of the intervention	1.75	2.69	5.27	Kruskal	0	Yes
HB	Pre-op hemoglobin	14.7	12.57	12.27	Kruskal	0.0119	Yes
HB	Post-op hemoglobin	11.75	11.03	11.28	Kruskal	0.497	Not
INR	Post-operative INR	1.88	2.47	1.3	Kruskal	0.3228	Not
INR	Pre-op INR	0.98	1.59	1.37	Kruskal	0.032	Yes
APTT	Pre-op APTT	24.28	27.7	31.15	Kruskal	0.0058	Yes
APTT	APTT post-op (sec)	21	26.41	32.04	Kruskal	0.025	Yes
FIBRINOGEN	Pre-op fibrinogen (mg/dL)	282.5	350.04	415.88	Kruskal	0.0013	Yes
FIBRINOGEN	Post-op fibrinogen (mg/dL)	311.32	375.89	476.46	Kruskal	0.2543	Not
GLU	Pre-op blood glucose (mg/dL)	132.5	103.39	151.27	Kruskal	0.0696	Not
GLU	Post-op blood glucose (mg/dL)	100.6	94.72	103.43	Kruskal	0.6449	Not
CREATE	Pre-op creatinine (mg/dL)	0.85	1.37	1.11	Kruskal	0.8367	Not
CREATE	Post-op creatinine (mg/dL)	2.07	2.59	2.68	Kruskal	0.7822	Not
CK-MB	CK-MB post-op (UI/L)	11.9	13.55	46.99	Kruskal	0.0354	Yes
CK-MB	CK-MB pre-op	7.88	14.77	18.37	Kruskal	0.064	Not

DM = diabetes mellitus; HT = hypertension; CRF = chronic renal failure; HB = hemoglobin; GLU = glucose; CREATE = creatinine; APTT = activated partial thromboplastin time; INR = international normalized ratio.

**Table 2:** Performance of the Random Forest model in predicting LOS.

	Precision	Recall	F1 Score	Support
Short hospitalization	1	0.667	0.8	9
Average hospitalization	0.75	0.75	0.75	12
Long hospitalization	0.75	1	0.857	9
Accuracy *	0.8	0.8	0.8	0.8
AVG Macro	0.833	0.806	0.802	30
weighted avg	0.825	0.8	0.797	30

* Accuracy: represents the total proportion of correct classifications made by the model, in relation to all the cases analyzed. In this study, an accuracy of 80% means that four out of five patients were correctly classified in the real category of hospitalization.

**Table 3:** Distribution of hospital length of stay (LOS) categories by age band.

Age_Band	Short	Medium	Prolonged
<70	10	83.3	6.7
70–79	3.7	51.9	44.4
>80	0	57.1	42.9

**Table 4:** Model comparison summary.

Model	Accuracy	F1 (Short)	F1 (Medium)	F1 (Long)	F1 (Macro Avg)	F1 (Weighted)
Random Forest	0.7	0	0.791	0.533	0.441	0.661
Logistic Regression	0.6	0	0.7	0.444	0.381	0.577
KNN	0.633	0	0.756	0.308	0.354	0.571

**Table 5:** Comparative Table of Prediction Models.

Characteristics	RAPT Score [6]	Ramkumar et al. [7]	H.I.P.P.O. Score
Type of model	Simple clinical score	ML algorithm (naïve Bayes)	Interpretable ML-based custom score
Surgical procedure analyzed	Total hip/knee arthroplasty	Primary total hip arthroplasty	Primary total hip arthroplasty
Number of patients	Not specified; validated in various populations	122,334 patients	Single-center cohort
Type of data used	Basic clinical data	Preoperative administrative data	Clinical + biological + organizational data
Included variables	Age, sex, mobility, social support, independence	Age, sex, race, comorbidity score, illness severity	HB, INR, glucose, APTT, fibrinogen, surgery day, CKMB, etc.
Model purpose	Predicting discharge disposition and LOS	Predicting LOS and reimbursement (PSPM)	Predicting hospital LOS
Performance (accuracy/AUC)	Variable accuracy; limited for intermediate scores	LOS: AUC = 0.87; accuracy = 82.9%	High accuracy (F1/precision/recall reported per class)
Clinical applicability	Very easy to use	Scalable in large systems, less interpretable	Easy to apply in non-digital hospital settings
Technical requirements	Minimal (can be applied on paper)	Moderate—requires database access and ML coding	Low—based on routinely collected clinical data

## Data Availability

The data presented in this study are available on request from the corresponding author due to privacy and legal reasons.
